# The most dreadful mushroom toxins: a review of their toxicological mechanisms, chemical structural characteristics, and treatment

**DOI:** 10.17179/excli2024-7257

**Published:** 2024-05-28

**Authors:** Irene Gouvinhas, Jani Silva, Maria José Alves, Juliana Garcia

**Affiliations:** 1 CITAB - Centre for the Research and Technology of Agro-Environment and Biological Sciences/ Inov4Agro - Institute for Innovation, Capacity Building and Sustainability of Agri-Food Production, University of Trás-os-Montes e Alto Douro, 5001- 801 Vila Real, Portugal; 2 AquaValor - Centro de Valorização e Transferência de Tecnologia da Água - Associação, Rua Dr. Júlio Martins n.º 1, 5400-342 Chaves, Portugal

**Keywords:** mushroom toxins, toxicological mechanisms, chemical structures, treatment

## Abstract

Mushroom consumption is a worldwide custom that continues to grow in popularity. On the other hand, foraging for wild mushrooms can lead to serious disease and even death if deadly mushrooms are accidentally consumed. Mushroom poisoning is difficult to diagnose and treat since the symptoms are similar to those of other disorders. In terms of chemistry, mushroom poisoning is associated with extraordinarily strong toxins, meaning that isolating and identifying toxins has substantial scientific relevance, especially in understanding the lethal components of toxic mushrooms. Most of these toxins exhibit exceptional physiological features that might help enhance chemistry, biochemistry, physiology, and pharmacology research. Despite the discovery of more than 100 poisons, several dangerous mushrooms remain unexplored. This review covers the chemistry (including chemical structures, complete synthesis, and biosynthesis), as well as the toxicology, namely the toxicokinetics, mechanisms of toxicology, and clinical toxicology of these poisons, in addition to the discussion of the development of their most effective diagnostic and therapeutic strategies with the hopes of spurring additional studies, focusing on individual classes of toxins found in poisonous mushrooms such as amatoxins, gyromitrin, orellanine, and phallatoxins.

See also the graphical abstract(Fig. 1).

## Abbreviations and Symbols

Ac-MF: 1-acetyl-2-methyl-2-formyl hydrazine

ACP: 1-aminocyclopropanol

ADH: Alcohol dehydrogenase

ALDH: Aldehyde dehydrogenase

ALF: Acute liver failure

CAT: Catalase

CCK-8: Cholecystokinin octapeptide

COX: Cyclooxygenase

DAO: Diamine oxidase

DNA: Deoxyribonucleic acid

ELISA: Enzyme-Linked Immunosorbent Assay

FDA: Food and Drug Administration

GABA: Gamma-aminobutyric acid

GI: Gastrointestinal 

GSH: Reduced glutathione

HPLC: High-performance liquid chromatography

HRP: Horseradish peroxidase

HTR: Head twitch response

H_2_O_2_: Hydrogen peroxide

IC_50_: Inhibitory concentration 

ICU: Intensive care unit

ID_50_: Infectious dose 50 %

IGluRs: Inhibitory glutamate receptors

i.p.: Intraperitoneal

i.v.: Intravenous

KO mice: knockout mice

LD_50_: Lethal Dose, 50 %

LT: Liver transplantation 

LSD: d-lysergic acid diethylamide

LPS: Lipopolysaccharide

MARS: Molecular Adsorbent Recirculating System

MFH: Methyl-formylhydrazine

MMH: Monomethyl-hydrazine

MMP: Mitochondrial membrane potential

mRNA: Messenger RNA

NAC: N-acetylcysteine

NAD: Nicotinamide adenine dinucleotide

NMFA: N-nitroso-N-methylformamide

OATP: Organic anion-transporting polypeptide 

OR: Orellanine

p.o.: Per os

RIA: Radioimmunoassay

RNA: Ribonucleic acid 

ROS: Reactive oxygen species

SOD: Superoxide dismutase

SSA: Anion radical scavenging activity

TNF: Tumor necrosis factor

US: United States

## Introduction

**Figure 2:**
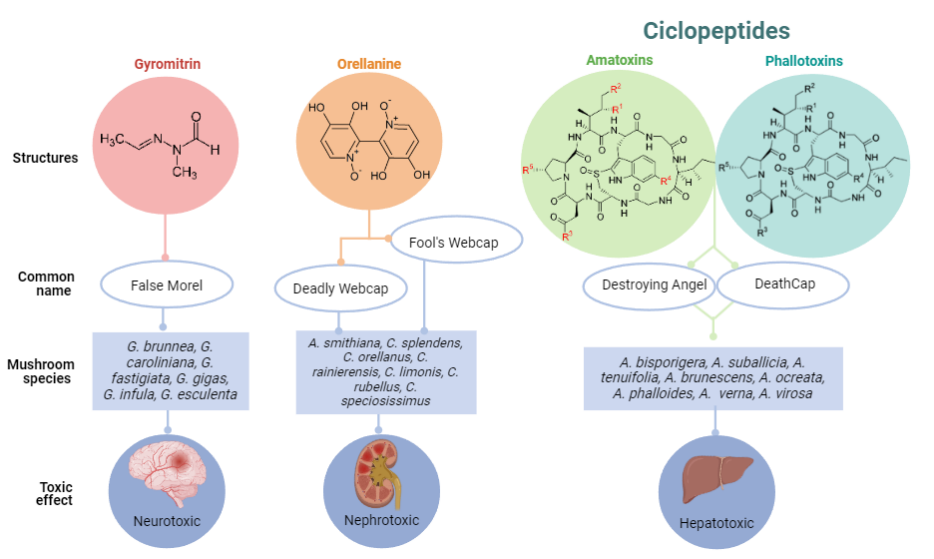
Main toxin groups synthesized by Mushrooms

Mushrooms belong to the higher phyla Ascomycota or Basidiomycota and have fleshy, spore-bearing fruiting bodies that grow above ground, on soil, or in their food supply (Cheung, 2008[23]). They are an excellent source of different and distinct bioactive secondary metabolites, which have a variety of therapeutic characteristics for a wide range of diseases (Garcia et al., 2021[45]). Edible species are a major source of carbohydrates, dietary fiber, and proteins for the general population, containing a great taste and low calories. In this context, wild-growing mushrooms have been widely consumed as a delicacy, particularly for their exquisite flavor and texture (Kalač 2009[56]). However, as wild mushrooms have become more popular, mushroom poisoning incidents have also been observed. Poisonous mushroom species contain one or more toxins, which may be classified based on their chemical structure and respective clinical toxicology. In 2019, they were divided into six classes based on their essential clinical features: (1) cytotoxic mushroom poisoning; (2) neurotoxic mushroom poisoning; (3) myotoxic (rhabdomyolysis) mushroom poisoning (4) metabolic, endocrine, and related toxicity mushroom poisoning; (5) gastrointestinal irritant mushroom poisoning; and (6) miscellaneous adverse reactions to mushrooms (White et al., 2019[117]). Among these, cytotoxic and neurotoxic mushrooms are the most dreadful mushrooms containing amatoxins, orellanine, and gyromitrin toxins (Figure 2(Fig. 2)). Toxic symptoms usually start in the gastrointestinal system and progress to organ damage and eventually death. Since most of these mushrooms contain powerful poisons, all these groupings have similar poisoning symptoms (Garcia et al., 2015[42]).

Structure identification, clinical toxicity, detection, early diagnosis from blood/urine, therapies, and usage in pharmaceuticals or other research fields have all been intensively explored for some of the reported mushroom poisons. Among them, the most dreadful toxins belong to cyclopeptides, gyromitrin, and orellanine groups and most of these poisons' harmful mechanisms remain unknown (Diaz, 2005[28]).

This review aims to examine the poisons produced by various mushrooms by examining the available experimental and clinical toxicology, focusing on the toxicity pathways. Furthermore, due to the inevitability of a poor prognosis in most intoxicated patients, special emphasis will be devoted to the amatoxins.

## Gyromitrin Poisoning

There are several toxic gyromitrin-containing mushroom species involved in gyromitrin poisoning, such as *Gyromitra esculenta*, commonly known as the “Beefsteak morel”, *Gyromitra montana*, commonly known as “The Walnut” and *Verpa bohemica*, commonly known as the “Early Morel” (Beug 2011[13]). These types of mushrooms are widely consumed since they have been attractive to hunters and gourmets due to their texture and taste, and it has been often harder to persuade individuals to not eat any of these species. The gyromitrin-containing mushrooms toxicity is mainly associated with the method of culinary preparation, demanding several steps to remove gyromitrin from mushrooms before their consumption. 

### Toxin 

Gyromitrin (see Figure 2(Fig. 2)) has the chemical formula C_4_H_8_N_2_O and is named N'-ethylidene-N-methylformohydrazide (Horowitz and Horowitz, 2017[53]). Since gyromitrin has a boiling point of 143 ºC (289 ºF) and is water-soluble and volatile, a thorough boiling or long-term drying is necessary to partially reduce the toxicity of gyromitrin-containing mushrooms (Michelot and Toth, 1991[72]). Gyromitrin when consumed is converted into toxic metabolites N-methyl-N-formylhydrazine (MFH) and N-methylhydrazine (MMH).

### Toxicity

Acute oral toxicity (LD_50_) of gyromitrin is markedly variable in different animal models: 344 mg/kg in mice (Wright et al., 1978[119]), and 320 mg/kg in rats (Mäkinen et al., 1977[69]), whereas in rabbits it varies from 50 mg/kg (Pyysalo, 1975[85]) to 70 mg/kg (Mäkinen et al., 1977[69]). Chickens, otherwise, seem to be insensitive to gyromitrin intoxication since no toxic effects were detected with doses of 400 mg/kg (Mäkinen et al., 1977[69]). The rabbits' and rats' poisoning symptoms included convulsions, hypersensitivity, loss of activity, lack of appetite, and severe weight loss intoxication. Rabbit's necropsy showed extensive fatty degeneration of the liver, but this effect was much less severe in rats. The human LD_50_ of gyromitrin has been evaluated as 20-50 mg/kg for adults and 10-30 mg/kg for children. These doses correspond approximately to 0.4-1 kg and 0.2-0.6 kg of fresh *G. esculenta* (Michelot and Toth, 1991[72]).

### Toxicokinetics of gyromitrin

The toxicokinetics of gyromitrin data on biotransformation are scarce. Toxicosis can result from oral and inhalation exposure. Ingestion of gyromitrin (acetaldehyde N-methyl-N-formylhydrazone)-containing mushrooms results in the hydrolysis of gyromitrin to MFH, which is further metabolized to MMH. The degree of hydrolysis is dependent on the pH in the stomach, but it is not complete (Wright et al., 1978[117]). Inhaling vapors during the cooking process can also result in poisoning. Hydrazines are further converted in the liver to reactive intermediates such as methyl cations and free methyl radicals.

### Mechanisms of toxicity

**Figure 3:**
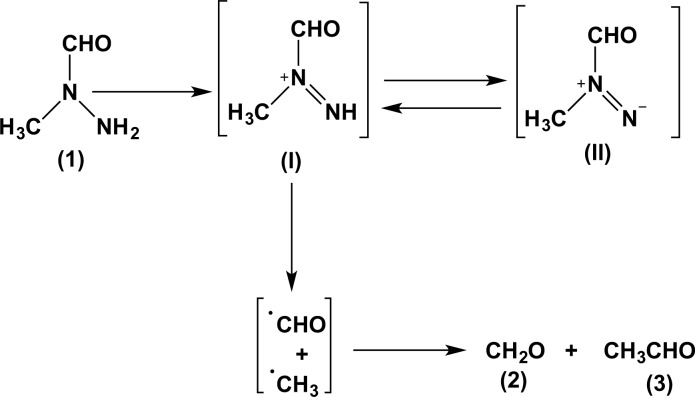
Intermediates (I, II) and products formed by oxidation of 1. The microsome-mediated oxidation of 1 yielded formaldehyde (2) and acetaldehyde (3)

Once ingested, gyromitrin is rapidly hydrolyzed in the stomach (acidic conditions), forming easily absorbed hydrazines MFH and MMH (Michelot and Toth, 1991[72]). Whereas the exact mechanism of toxicity remains unknown, several studies have demonstrated many effects in multiple organ systems. The oxidation of MFH was investigated to determine the intermediates that may develop during metabolism (Gannett et al., 1991[38]). MFH oxidation yielded formaldehyde and acetaldehyde. The formation of acetaldehyde requires the oxidation of MFH to a diazenium ion (I) or diazene (II) and fragmentation of I/II to formyl and methyl radicals (Figure 3(Fig. 3)) (Gannett et al., 1991[38]). 

Moreover, MFH is a non-competitive inhibitor of human intestinal diamine oxidase (ID_50_ = 1.6 x10^-5^ mol/L), this concentration corresponding to less than 5 g of fresh mushroom. Noteworthy, strongly decreased intestinal diamine oxidase (DAO) activity has been linked to intestinal disorders with the proliferation of the mucosa (Biegński et al., 1984[16]).

Another enzyme that is directly inhibited by gyromitrin is the pyridoxal phosphokinase. This enzyme is responsible for dietary vitamin B6 (pyridoxine) conversion into active pyridoxal 5-phosphate (Horowitz et al., 2024[53]). Moreover, *in vitro *and *in vivo*, MMH may generate hydrazones with pyridoxal-5-phosphate (Barceloux, 2008[8]). Pyridoxal-5-phosphate is a cofactor for glutamic acid decarboxylase and GABA transaminase in the gamma-aminobutyric acid (GABA) synthetic pathway, which results in decreased GABA synthesis (Barceloux, 2008[8]). MMH can directly block glutamic acid decarboxylase when given intraperitoneally to rats at a concentration of 0.8 mM/kg, resulting in a further decrease in GABA levels (Medina 1963[71]). Hydrazones may also cause lipid peroxidation in the liver, leading to cytotoxicity and acute liver damage. Hydrazones might potentially act as oxidizing agents, causing methemoglobinemia. Although methemoglobin production is frequently discussed as a potentially harmful consequence of gyromitrin mushroom ingestion in humans, there is just one case record in a dog (Horowitz et al., 2024[53]).

MMH toxicity has also been linked to the formation of free radical intermediates that produce glutathione depletion in isolated hepatocytes and liver microsomes (Albano et al., 1989[1]).

### Carcinogenicity

MMH, (Toth and Shimizu 1973[110]) as well as its precursors MFH (Toth and Patil, 1979[106], 1982[107]; Toth et al., 1979[108]) and raw *G. esculenta* (Toth et al., 1992[109]), have been shown to be carcinogenic in *in vivo* studies. *G. esculenta,* when administered orally to Swiss mice (6 weeks), could induce tumors in the lungs, nasal cavity, blood vessels, forestomach, glandular stomach, cecum, and liver (Toth et al., 1992[109]).

The MMH administration of 0.01 % solution in the drinking water to 6-week-old Syrian golden hamsters (lifetime) was able to generate malignant histiocytomas of the liver and cecum tumors (Toth and Shimizu, 1973[110]). In mice, MFH has also been shown to be tumorigenic to several organs (Toth and Patil, 1982[107]; Toth et al., 1979[108]). Similarly, MFH administration in drinking water as a 0.0039 % solution to randomly bred Swiss albino mice (six weeks old) throughout life was able to induce tumors in the lungs, livers, blood vessels, gall bladder, and bile ducts. This effect was also observed in Syrian golden hamsters, with MFH causing the formation of benign and malignant liver cell tumors, malignant histiocytomas, and tumors of the gallbladder and bile ducts after administration of 0.0078 % MFH in drinking water (Toth and Patil, 1979[106]). MFH was also shown to be mutagenic in the AMES assay, especially in the presence of a metabolic activation system (Von Der Hude and Braun, 1983[114]). It is suggested that the oxidation of MFH to a diazenium ion (I) or diazene (II) intermediates may be important in understanding and elucidating carcinogenesis by MFH (Gannett et al., 1991[38]). However, in humans, no carcinogenic effects were reported since there are no case reports and/or epidemiological studies in the literature, which is probably due to the sporadic consumption of this mushroom.

### Clinical toxicology

Initial clinical symptoms are associated with gastrointestinal (GI) and neurological disorders. Nausea, vomiting, headache, abdominal pain, and occasionally watery or bloody diarrhea and hypoglycemia may occur after a latency period of 6-8 hours. In most cases, the poisonings are limited to gastrointestinal disorders, and the patient recovers 2 or 5 days later without any after effects (Michelot and Toth, 1991[72]). In severe cases of intoxication, neurological disorders, such as nervousness, delirium, coma, and convulsions, complete the clinical condition (Michelot and Toth, 1991[72]). It may, rarely, occur epileptogenic neurotoxicity, with vertigo, delirium, seizures, stupor, and coma, especially in the elderly and in patients on isoniazid prophylaxis or treatment for tuberculosis (Diaz, 2005[28]). Hepatic and renal damage are potential complications of gyromitrin poisoning and may be fatal in 2-4 % of cases (Barceloux, 2008[8]; Singh et al., 2019[99]). 

### Analytical methodology

In the literature, only a few quantitative analytical methodologies for gyromitrin have been published. Older methods for the evaluation of gyromitrin in mushrooms were based on thin-layer chromatography (TLC) and spectrofluorometry (gyromitrin and MMH) (Andary et al., 1984[3]). However, most of the studies use gas chromatography (GC-MS) to determine gyromitrin and MMH levels in mushrooms. The method consists of determining the total hydrazones content based on acid hydrolysis of gyromitrin and other related hydrazones in air-dried *Gyromitra esculenta* followed by derivatization of MMH with pentafluorobenzoyl chloride and subsequent determination by GC-MS (Arshadi et al., 2006[6]). MMH was detected in mice peritoneal fluid by gas-liquid chromatography (Von Wright et al., 1978[115]) and in rat serum by high-performance liquid chromatography (HPLC) with electrochemical detection (Slanina et al., 1993[100]). Gyromitrin was determined using glass-capillary gas chromatography and NMR spectroscopy (Mäkinen et al., 1977[69]) and there is a report of post-mortem examinations of gyromitrin in viscera by IR and UV spectroscopy and TLC (Giusti and Carnevale, 1974[46]). More recently, a theoretical description has been performed for the determination of gyromitrin through an electrochemical methodology. In this view, Tkach et al., (2019[104]) concluded that vanadium (III) oxyhydroxide may be efficient in the determination of gyromitrin. For that, the electroanalytical process is performed cathodically with a gradual reduction of the products, becoming an efficient and diffusion-controlled method for gyromitrin determination (Tkach et al., 2019[104]).

### Diagnosis and treatment 

The recognition of gyromitrin-containing mushrooms in meals aids in the diagnosis of gyromitrin toxicosis. To distinguish between the authentic and the fake morels, a mycologist's identification is required. Because detecting gyromitrin, hydrazine analogs, or metabolites in mushrooms or biological materials is not regularly accessible, diagnosis is based mostly on clinical and clinicopathological symptoms, as well as mushroom identification (Puschner, 2018[83]).

After exposure to gyromitrin, most people only have minor gastrointestinal problems and recover completely within a few days. As of the late onset of clinical symptoms, early decontamination is sometimes impossible. Activated charcoal has been advocated for treatment, despite the lack of effectiveness evidence (Karlson-Stiber and Persson, 2003[57]). In cases of severe central nervous system toxicity, it is relevant to give pyridoxine (25 mg/kg or 5 g intravenous dose up to a daily adult dose of 15 - 20 g) since it counteracts the inhibition of GABA synthesis (Barceloux, 2008[8]). The administration of benzodiazepines, such as lorazepam is also used in case of seizures. Administration of folinic acid and N-acetylcysteine has been recommended in humans, however, controlled studies have not been performed, and thus their relevance remains to be established (Puschner, 2012[84]).

## Orellanine

Orellanine is a potent nephrotoxin found in some species of the genus *Cortinarius*, such as *Cortinarius orellanus* and *C. speciosissimus,* which was first isolated in 1962 (Grzymala, 1962[47]) and then in 1975, the chemical structure was proposed (Antkowiak and Gessner, 1975[4]). These authors identified another compound orellinine, which was suggested to be the monodeoxo derivative of orellanine (Antkowiak and Gessner, 1975[4]). Orellanine is a colorless, finely crystalline, blue-fluorescing chemical that decomposes slowly when heated over 150 ºC and exposed to UV radiation to the yellow, nontoxic bipyridyl product orelline (Antkowiak and Gessner, 1979[5]; Barceloux, 2008[8]). The three-dimensional structure of the toxin was determined using X-ray crystallography by Cohen-Addad et al., in 1987. Orellanine and orelline are practically insoluble in organic solvents and water, being soluble in methanol, pyridine, dimethyl sulfoxide (DMSO), and trifluoroacetic acid (TFA) (Antkowiak and Gessner, 1979[5]). Cooking, freezing, or drying do not affect the toxicity of orellanine (Karlson-Stiber and Persson, 2003[57]).

### Toxicity

The content of orellanine was determined in dried fungus and the amounts range from 9 mg/g in *C. speciosissimus *to 14 mg/g in *C. orellanus* (Karlson-Stiber and Persson, 2003[57]). There are significant inter- and intra-species differences in the toxic reaction to orellanine, according to the literature. In mice, the intraperitoneal and oral LD_50_ are respectively 12.5 and 90 mg/kg (Richard et al., 1988[89]), however, in another study the intraperitoneal and oral LD_50_ are 15 and 33 mg/kg, respectively (Prast et al., 1988[82]). Oral administration of *C. orellanus* mushrooms to male Sprague Dawley rats resulted in a LD_50_ of 967 mg/kg body weight. The gender also appears to influence the toxicity since rat females seem to be more resistant to oral orellanine (Nieminen and Pyy, 1976[75]). Likewise, in humans, the inter-individual variability is also confirmed (Danel et al., 2001[25]). The human lethal dose of fresh mushrooms was estimated to be 100 - 200 g (Grzymala, 1957[48]). According to animal research, a 70-kg person's deadly dosage would be about 7.5 mg/kg of body weight, which would be found in roughly 70 g of dry mushrooms or 700 g of fresh mushrooms (Raff et al., 1992[86]).

### Toxicokinetics of orellanine

The toxicokinetics of orellanine in humans are not fully understood since samples are not obtained until several days after ingestion due to the long latent period before the onset of illness (Dinis-Oliveira et al., 2016[30]). After oral administration of 2 g of *C. orellanus* to male Sprague Dawley rats, detectable levels of orellanine were excreted only during the first 24 hours (Prast et al., 1988[82]). In another *in vivo *study using rats, orellanine was almost exclusively eliminated by glomerular filtration as well as by peritoneal dialysis (Najar et al., 2018[74]). In human cases of orellanine poisoning, the toxin was not detected in urine samples collected from 2 to 18 days after ingestion (Dinis-Oliveira et al., 2016[30]). According to Rohrmoser et al. (1997[92]), orellanine and its decomposition products cannot be detected in urine, plasma, and dialysis fluids after the onset of symptoms, as most of the toxin is concentrated in the kidneys at this time (Rohrmoser et al., 1997[92]). Orellanine was qualitatively detectable in small quantities of renal biopsy samples taken 9 and 60 days after the mushroom ingestion, which suggests a relatively long period after the onset of intoxication (Rohrmoser et al., 1997[92]). 

### Mechanism of toxicity

**Figure 4:**
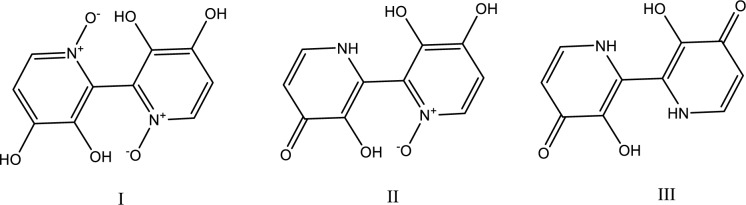
Chemical structure of orellanine (I), orellinine (II), and orelline (III)

The toxicity mechanism of orellanine is not yet fully understood. Orellanine is reported to be converted to orellinine, which is then reduced to harmless orelline via photochemical degradation (Figure 4(Fig. 4)). Orellanine's chemical structure is similar to that of the pyridine herbicides diquat (1,1'-ethylene-2,2'-bipyridinium) and paraquat (1,1'-dimethyl-4,4'-bipyridinium), leading some researchers to propose a monoelectronic reduction process (generation of a stable radical) (Cantin et al., 1988[22]; Richard et al., 1987[90]). However, an *in vivo* analysis of orellanine's electrochemical behavior indicates that its mode of action must be distinct from that of other poisons. However, because orellanine has a substantially higher negative redox potential than paraquat and diquat, this hypothesis has been questioned. Orellanine disrupts LLC-PK1 cell monolayers and inhibits membrane-bound alkaline phosphatase and cytosolic lactate dehydrogenase activity. Orellanine's intracellular mechanism of action is confirmed by the fact that the cell membrane remains undamaged even after the monolayer disruption has begun (Heufler et al., 1987[51]). When LLC-PK1 monolayer and Caco-2 cell cultures were treated with orellanine a complete inhibition of alkaline phosphatase activity could be observed after 24 hours of incubation with 1 mM orellanine (Ruedl et al., 1989[94]). Kinetic studies were performed with renal, intestinal, and placental alkaline phosphatase isoenzymes using different cell homogenates to investigate the specific inhibitory actions of orellanine. Orellanine acts on renal alkaline phosphatase of LLC-PK1 cells as well as on bovine kidney preparation as a non-competitive inhibitor and as a competitive inhibitor on intestinal alkaline phosphatases from Caco-2 cell and bovine intestinal mucosa preparation as well as placental isoenzyme (Ruedl et al., 1989[94]). A study using the electron spin resonance (ESR) technique supported the participation of radicals in the toxicity mechanism (Richard et al., 1995[88]). Richard et al. (1995[88]) observed the formation of a radical form of orellanine identified as *ortho*-semiquinone anion radical which was generated by oxidation of orellanine through a variety of methods, including photooxidation (visible and UV oxidation), chemical [hydrogen peroxide (H_2_O_2_)], biochemical (e.g cytochrome C), and enzymatic oxidation (mushroom tyrosinase-O_2_ system) (Richard et al., 1995[88]). The *ortho*-semiquinone anion radical is responsible for superoxide hydrogen peroxide, and hydroxyl radicals production which reduces glutathione (GSH). Thereby, oxidative stress seems to be the main orellanine toxic mechanism which may lead to large oxygen consumption, creating hypoxic conditions, as well as depletion of renal glutathione level (Richard et al., 1995[88]). Corroborating this hypothesis, ESR and spin-trapping studies, demonstrate that orellanine can be oxidized to an *ortho*-semiquinone radical intermediate by horseradish peroxidase (HRP) and the H_2_O_2_ system (Oubrahim et al., 1998[78]). Furthermore, the production of ascorbyl or glutathionyl radicals was seen when orellanine was oxidized by HRP/ H_2_O_2_ in the presence of reducing agents of biological importance such as ascorbic acid or glutathione (Oubrahim et al., 1998[78]). To support these findings an *in vivo* study was performed to clarify the mechanisms behind the kidney damage. Sprague-Dawley rats, received various doses of intraperitoneal doses of orellanine (0-5 mg/kg). One week later, renal function (GFR), ascorbyl radicals, oxidative protein damage, renal mRNA levels, catalase, glutathione peroxidase, and superoxide dismutase were evaluated. It was evident that orellanine caused a dose-dependent decrease in GFR, paralleled by increased levels of ascorbyl radicals and oxidative protein damage. In addition, the expression of mRNA for antioxidative enzyme-related genes was strongly decreased. These data strongly suggest that orellanine nephrotoxicity *in vivo* is mediated by oxidative stress, including a virtual shutdown of important antioxidative enzymes (Nilsson et al., 2008[76]). 

### Clinical toxicology

Orellanine poisoning is characterized by a long latency period (2-6 days) before the onset of the disease. It follows a prerenal gastrointestinal phase, which is characterized by vomiting, nausea, and diarrhea. Other signs may coexist such as asthenia, lumbar pain, anorexia, thirst, chills, headache, and myalgia. Clinical signs may spontaneously disappear in some patients, leaving the poisoning unnoticed; in others, the signs become more intense and are accompanied by neurological manifestations (paraesthesia, taste impairment, cognitive disorders), lumbar pain, and anuria requiring hospitalization (Bouget et al., 1990[18]). The hallmark of orellanine toxicity is acute renal failure which is accompanied by several renal damage symptoms such as lumbar and flank pains, intense thirst, oliguria, and polyuria (Danel et al., 2001[25]; Dinis-Oliveira et al., 2016[30]). Early and severe interstitial fibrosis, interstitial edema, and tubular epithelial necrosis are the most common features of renal lesions (Duvic et al., 2003[31]). The incidence of acute renal failure varies from 30 to 46 % and it depends on individual susceptibility, pre-existing renal disease, and the cumulative dose of toxin ingested (Duvic et al., 2003[31]), and in 50 % of the cases may progress to chronic renal insufficiency (Flesch and Saviuc; 2004[36]). Moreover, renal failure occurs 4 - 15 days (median: 8 days) after ingestion depending on the dose ingested (Barceloux; 2008[8]). A renal biopsy performed on two patients showed pronounced focal tubular damage with tubulorrhexis, cast formation, and severe interstitial edema with patchy infiltration of lymphocytes, plasma cells, and some polymorphs (Short et al., 1980[98]). Another kidney biopsy obtained from 5 patients performed 10-21 days after fungal ingestion showed a similar pattern dominated by acute tubular necrosis, tubular dilatation, tubular cellular atrophy and degeneration, apoptotic bodies, interstitial edema, and focal inflammation but no eosinophilia (Hedman et al., 2017[49]). Liver injury has also been shown based on the analysis of transaminases and bilirubin levels, as well as hepatomegaly, hepatalgia, and necrosis lesions, nevertheless, most of the studies ruled out liver involvement (Dinis-Oliveira et al., 2016[30]). 

### Analytical methodology

Orellanine has been isolated and analyzed using a variety of analytical methods. A color test based on the ferric-orellanine reaction has been developed for routine identification of the toxin in mushroom samples. A fresh or dried mushroom is crushed in five volumes of water and filtered after 10 min at room temperature. The filtrate is then mixed with an equal amount of 3 % ferric chloride hexahydrate dissolved in 0.5 N hydrochloric acid (HCl). The presence of orellanine is suspected if a dark gray-blue color ink appears (Dinis-Oliveira et al., 2016[30]). Quickly and simple analytical methods are available to detect orellanine using TLC on silica-gel or cellulose plates which allow for analysis of the toxin in mushroom or tissue samples (Rapior et al., 1989[87]; Rohrmoser et al., 1997[92]). Other analytical procedures are available such as electron spin resonance spectroscopy (ESR) analysis which allowed the detection of the oxidation product of orellanine (Oubrahim et al., 1997[79]). HPLC methods with electrochemical (Holmdahl et al., 1987[52]), UV (Cantin et al., 1989[21]), and diode-array (Koller et al., 2002[60]) detectors have also been described. These HPLC methods were developed to analyze orellanine in mice serum directly (Holmdahl et al., 1987[52]) and mushrooms (Cantin et al., 1989[21]; Koller et al., 2002[60]). Additionally, an HPLC-electrospray tandem mass spectrometry method for the analysis of total orellanine in mushrooms and plasma is also available in the literature (Herrmann et al., 2012[50]). Moreover, to analyze orellanine in tissues, HPLC and LC-MS/MS methods were developed (Srivastava et al., 2016[101]).

Alternatively, a more sensitive GC-MS method can be used for the analysis of orellanine in biological samples (Brondz et al., 2012[20]). The direct identification of orellanine in the stomach contents of rats after they were fed food containing *C. orellanus* was achieved using GC-MS with supersonic molecular beams (SMB) (Brondz et al., 2012[20]). According to the authors, the GC approach gives much greater separation of compounds in a combination than TLC or HPLC, as well as higher repeatability (Brondz et al., 2012[20]).

### Diagnosis and treatment

Unfortunately, so far, there is no known antidote against orellanine poisoning. Principles of treatment include limiting absorption, enhancing elimination, correcting metabolic abnormalities, and providing supportive care (Barceloux, 2008[8]; Danel et al., 2001[25]; Dinis-Oliveira et al., 2016[30]). The decontamination of poisoned patients using emesis or gastric lavage is useful if the patients seek medical care earlier than 6 hours after ingestion (Dinis-Oliveira et al., 2016[30]). Early secondary decontamination by either plasmapheresis or hemoperfusion has no proven benefit in removing orellanine and preventing renal failure after *Cortinarius* ingestions (Diaz, 2005[28]). As the kidneys seem to be the target organ of orellanine poisoning a periodic accurate assessment of changes in renal function is crucial for monitoring the renal function. At the late stage of severe chronic renal insufficiency, the renal transplant is a last resort treatment measure (Danel et al., 2001[25]). Renal transplantation should not be performed too early in the course of illness, as complete renal recovery may occur in 40-60 % of *Cortinarius*-poisoned patients (Diaz, 2005[28]). As the orellanine mechanism of toxicity seems to involve reactive oxygen species with large oxygen consumption and depletion of glutathione some authors recommend the use of N-acetylcysteine, corticosteroids, and selenium (Kilner et al., 1999[59]; Wörnle et al., 2004[118]). In fact, Kerschbaum et al. (2012[58]) reported a case of a couple with acute renal failure after accidental intake of *C. rubellus *and a favorable outcome after treatment with high-dose antioxidant therapy with N-acetylcysteine and steroids. Thereby, the authors suggested that early treatment with antioxidant therapy and steroids might be effective in reducing the risk of developing end-stage renal failure (Kerschbaum et al., 2012[58]). However more studies are needed to strengthen this conclusion.

## Cyclopeptides

Toxic cyclopeptides are usually found in many mushrooms, such as *Amanita bisporigera, Amanita phalloides, Galerina marginata, Lepiota castanea*, and several other mushrooms that might be mistaken for edible mushroom species (Schneider, 2014[95]). The cyclopeptides consist of two principal peptides: amatoxins and phallotoxins (Schneider 2014[95]). Amatoxins and phallotoxins are bicyclic, cross-linked by the 2'-bound sulfur group (Vetter 1998[112]).

### Phallotoxins

**Figure 5:**
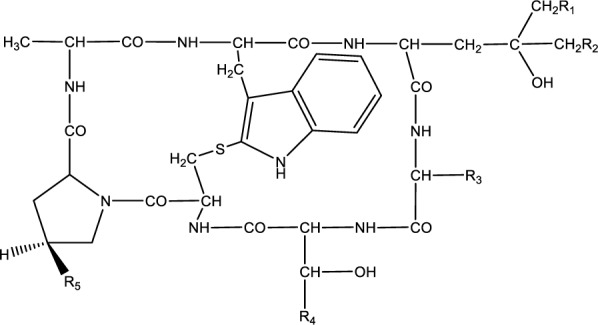
Chemical structure of phallotoxins R1=R5=OH, R2=H, R3=R4=CH3; Phalloidin R1=R2=H, R3=R4=CH3, R5=OH; Phalloin R1=R2= R5=H, R3=R4=CH3; Prophallin R1=R2= R5=OH, R3=R4=CH3; Phallisin R1=R2=H, R3=CH(CH3)2, R4=COOH, R5=OH; Phallacin R1=R5=OH, R2=H, R3=CH(CH3)2, R4=COOH; Phallacidin R1=R2=R5=OH, R3=CH(CH3)2, R4=COOH; Phallisacin

Phallotoxins are bicyclic heptapeptides that include phalloidin, phalloin, prophallin, phallisin, phallicin, phallacidin, and phallisacin (Figure 5(Fig. 5)) (Vetter, 1998[112]). Despite phallotoxin's high toxicity, these toxins are not able to be absorbed by the gastrointestinal tract, suggesting negligible effect in human poisoning. Therefore, the toxicity of cyclopeptide mushrooms is mostly due to the amatoxins, which will be the focus of the discussion in the following sections (Karlson-Stiber and Persson, 2003[57]).

### Amatoxins

**Figure 6:**
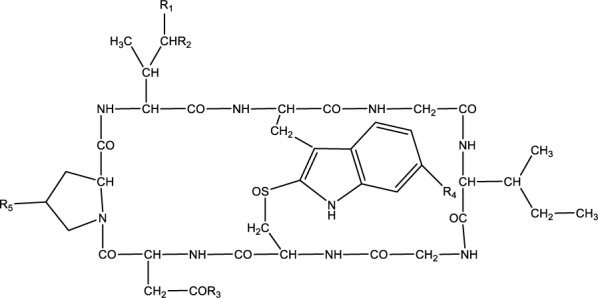
Chemical structure of Amatoxins R1=CH_2_OH, R2=R4=R5=OH, R3=NH_2_; α-Amanitin R1=CH2OH, R2=R3=R4=R5=OH; β-Amanitin R1=CH3, R2=R4=R5=OH, R3=NH2; γ-Amanitin R1=CH3, R2=R3=R4=R5=OH; ε-Amanitin R1=CH2OH, R2=R3=R5=OH, R4=H; Amanin R1=CH2OH, R2=R5=OH, R3=NH2, R4= H; Amanin amide R1=CH3, R2=H, R3=NH2, R4=R5=OH; Amanullin R1=CH3, R2=H, R3=R4=R5=OH; Amanullic acid R1=CH3, R2=R5=H, R3=NH2, R4=OH; Proamanullin

Amatoxins are bicyclic octapeptides and consist of nine defined members: α-amanitin, β-amanitin, γ-amanitin, ε-amanitin, amanin, amanin amide, amanullin, amanullinic acid, proamanullin (Vetter 1998[112]). From these, alpha and beta-amanitins predominate producing systemic toxicity (Schneider 2014[95]). Specific properties characterized these toxins: heat stability, water solubility, and resistance to enzyme degradation (Poucheret et al., 2010[80]). All groups of toxic peptides contain a tryptophan residue substituted at position 2 of the indole ring by a sulfur atom (Figure 6(Fig. 6)). 

Only the number of hydroxy groups and an amide carboxy exchange distinguish amatoxins. This group of toxins can be found in the *Amanita*, *Lepiota,* and *Galerina* species (e.g. *A. phalloides*, *A. virosa*, *A. verna*, *A. ocreata*, *A. bisporigera*, *A. suballiacea*, *A. tenuifolia*, and *A. hygroscopica*) (Karlson-Stiber and Persson, 2003[57]). From these*, A. phalloides* are responsible for the majority of fatalities caused by mushroom poisoning (Garcia et al., 2015[42]). It is the most toxic species which, apart from rare exceptions, contains 2 to 3 mg of amatoxins per gram of dry tissue. This mushroom is found growing all over central Europe and can easily be confused with the edible Horse Mushroom (*Agaricus arvensis*) and *Tricholoma equestre*. At an early development stage, an Amanita “button” can easily be mistaken for an edible puffball (*Lycoperdon bovista*) (Garcia et al., 2015[42]). 

#### Toxicity

There is a significant inter- and intra-species variation in the concentration of amatoxins in mushrooms, depending on several factors including growing conditions, time of year, and humidity (Garcia et al., 2015[44]). Therefore, accurate prediction of toxicity based on the number of mushrooms consumed is difficult. The lethal dose of amatoxins in humans could be estimated from accidental intoxications to be about 0.1 mg/kg body weight or around 7 to 8 mg of toxin (Baumann et al., 1993[9]) and this may be present in a single mushroom (Mas, 2005[70]). The first symptoms of intoxication are nausea, vomiting, and diarrhea, with hepatic injuries being more pronounced around 24-36 hours (Schneider, 2014[95]). In cases of intoxication with α-amanitin, patients can recover but, depending on the degree of injury, liver transplantation might be necessary (Schneider, 2014[95]). Since amatoxins are thermally stable, drying or boiling seems not to be sufficient to inactivate their toxicity (Baumann et al., 1993[9]).

#### Mechanism of toxicity

**Figure 7:**
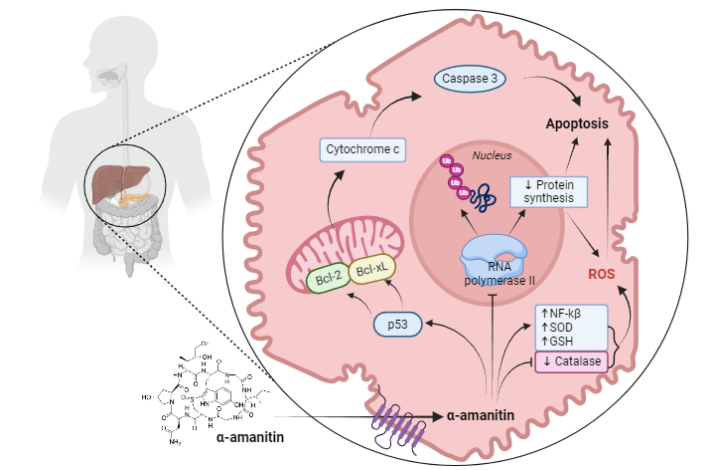
Main toxic mechanisms of amanitins within hepatocytes

Amatoxins are able to inhibit mainly the activity of the RNA polymerase II (RNAP II) and also polymerase III (RNAP III), through α-amanitin and β-amanitin (respectively) (Diaz 2018[27]), resulting in decreases in mRNA content, causing deficient protein synthesis and cell death (Garcia et al., 2015[42]) (Figure 7(Fig. 7)). Our previous findings suggest that α-amanitin may interfere with RNAP II transcription by interfering with the function of the trigger loop (TL) (Garcia et al., 2014[40]). The identified direct interactions between α-amanitin and TL residues Leu1081, Asn1082, Thr1083, His1085, and Gly1088 change the elongation process, contributing to RNAP II suppression. We further show that α-amanitin may bind directly with the Gly819, Gly820, and Glu822 bridge helix residues, as well as indirectly with His816 and Phe815 (Garcia et al., 2014[40]). The bridge helix is destabilized and RNAP II activity may be lost as a result. RNAP II has been demonstrated to be ubiquitinated and degraded by the proteasome as a result of this enzymatic inhibition, which has been linked to an increase in intracellular ATP concentrations (Rodrigues et al., 2020[91]).

The inhibition of RNAP II causes necrosis of eukaryotic cells being especially harmful to those cells that are in initial contact with the toxins (gastroenterological mucosa) and for those cells that have rapid turnover rates (liver and kidneys) (Bonnet and Basson, 2002[17]). 

The basic features of α-amanitin uptake have been well described already by Letschert et al. (2006[64]). The authors analyzed the transport of radioactively labeled O-methyl-dehydroxymethyl-α-amanitin into transfected MDCKII cells expressing human OATP1B3, OATP2B1, or OATP1B1 (Letschert et al., 2006[64]). The results indicated that only OATP1B3-expressing MDCKII cells took up labeled α-amanitin while the MDCKII cells expressing OATP2B1 and OATP1B1 showed insignificant transport activity. To obtain more evidence for OATP1B3 as the main uptake transport protein for amatoxins, the viability of cells transfected with the various hepatic OATP proteins was studied in the presence of amatoxin. Further support for amatoxin transport was found in primary human hepatocytes, expressing OATP1B3, OATP2B1, and OATP1B1, where cholecystokinin octapeptide (CCK-8), a specific substrate for OATP1B3, prevented the fragmentation of nucleoli, a typical lesion for amanitin action (Letschert et al., 2006[64]).

α-Amanitin has been shown to act synergistically with cytokines such as tumor necrosis factor-alpha (TNF-α) and this may be the final cause of liver failure. Leist et al., (1997[63]) provide convincing evidence that endogenous TNF-α and the induction of hepatocyte apoptosis are key phenomena in the hepatotoxicity of α-amanitin. For the *in vivo* toxicity of α-amanitin, the authors propose two alternative mechanisms of action. It may cause direct injury to parenchymal cells, which may both sensitize cells to cytokine assault and serve as the source of cytokine release. Second, it may promote cytokine overproduction by permitting LPS mobilization from the gut, encouraging the creation of local inflammatory foci, or by direct, yet undiscovered, contact with cytokine-producing cells. The final cause of liver failure may be the synergistic effect of endogenous cytokines such as TNF-α with xenobiotic-sensitized cells. 

The synergistic action of endogenous cytokines such as TNF-α with α-amanitin-sensitized cells may be the final cause of liver failure. El-Bahay et al. (1999[32]) found no evidence that amanitin toxicity is dependent on the presence of TNF-α in rat hepatocyte culture, as previously reported for mouse hepatocyte culture (Leist et al., 1997[63]). In rat hepatocytes, a concentration of 0.1 M amanitin, which is similar to that observed in intoxicated humans, was enough to cause cytotoxicity as well as RNA and protein synthesis inhibition. TNF-α at a subtoxic level of 50 ng/ mL did not affect amanitin-induced reduction of RNA and protein synthesis, but it did enhance the cytotoxic indications and shifted the onset of these symptoms earlier. Even after 36 hours, lipid peroxidation with α-amanitin alone is minimal, but cotreatment with TNF-α significantly increases it. The antioxidant silibinin blocks the action of TNF-α, suggesting that reactive oxygen species are involved. To summarize, rat hepatocyte culture responds to a clinically relevant dosage of amanitin by rapidly inhibiting RNA and protein synthesis, and TNF-α shortens the latency time and exacerbates cytotoxicity through a mechanism that may include reactive oxygen species (El-Bahay et al., 1999[32]). 

The *in vivo* effect of α-amanitin on superoxide dismutase (SOD), catalase activities (CAT) as well as the level of LPO products in liver homogenates has also been studied (Zheleva et al., 2007[120]). After incubation of the toxin with those enzymes, the results showed that α-amanitin may significantly boost SOD activity while significantly inhibiting CAT activity. The inhibition of substrate (H_2_O_2_) access to the CAT Fe-heme pocket might explain the *in vivo* α-amanitin inhibitory impact on CAT activity. *In vivo, *liver accumulation of the toxin may result in the production of reactive oxygen species (ROS), particularly O_2_ excess (Zheleva et al., 2007[120]). New mechanistic findings have revealed that amanitins stimulate the formation of GSH and tGSH, supporting the concept that oxidative stress plays a role in this pathogenesis (Rodrigues et al., 2020[91]).

Amanitin-induced stress signals activate the p53 protein, allowing it to form complexes with anti-apoptotic proteins (Bcl-XL and Bcl-2) and promote apoptosis via mitochondrial cytochrome c release into the cytosol (Garcia et al., 2015[42]). It has been discovered that α-amanitin causes changes in the mitochondrial proteome (Wang et al., 2018[116]). Indeed, according to a previous study, mitochondria may be a major subcellular location for α-amanitin at this toxin-reversible concentration (Wang et al., 2018[116]). 

According to our previous studies, NF-κB was shown to be highly activated in the liver and kidney after exposure to α-amanitin (Garcia et al., 2015[43], 2019[41]). For the first time, NF-κB factor was revealed to have a role in α-amanitin toxicity in those studies. Furthermore, NF-κB activation can induce liver damage through genetically regulating TNF-α and IL-6 expression. There are still numerous unknowns about amanitins' cellular toxicity, which explains the lack of very effective specialized therapy.

#### Toxicokinetics

Amatoxins are easily absorbed and distributed to target tissues 90-120 minutes after consumption. During the first 24-48 hours period following intake, amanitins are detectable in plasma at low quantities (peak values varied between 8 and 190 ng/mL) (Jaeger et al., 1993[55]). The absence of binding to plasma proteins explains the fast distribution at the hepatic and renal levels.

In human cases, amanitin was found in 11 of 43 amatoxin-intoxicated individuals who were exposed to amatoxin within 36 hours. The plasma values of α- and β-amanitins were 8-190 ng/mL and 15.9-162 ng/mL, respectively. In 24 of 35 patients, the total quantity of α- and β-amanitin excreted in urine was assessed and the concentrations were variable ranging from 0.03 to 3.29 mg for α-amanitin and 0.05 to 5.21 mg for β-amanitin (Jaeger et al., 1993[55]). Four cases of amatoxin-intoxicated individuals presented 10-19 ng/g liver and 122-1719 ng/g kidney for α-amanitin vs. 170.8-3298 ng/g liver and 1017-1391 ng/g kidney for β-amanitin (Jaeger et al., 1993[55]).

Faulstich et al. (1985[35]) also discovered that bile may remove a small amount of toxins (approximately 7 %), which can then be reabsorbed at the intestinal level, completing the enterohepatic cycle. Furthermore, these same authors observed that α-amanitin was eliminated from the feces during the first 24 hours (Faulstich et al., 1985[35]). 

#### Clinical toxicology

Four distinct phases of *A. phalloides* intoxication have been identified in the literature: the latent period (6-48 hours, phase 1) is followed by the gastrointestinal phase (12-24 hours, phase 2), which is characterized by vomiting, diarrhea, and abdominal discomfort. The shorter the delay in the first symptom occurrence, the greater the toxicity (Lecot et al., 2023[62]; Schneider, 2014[95]). Following these symptoms, there is a second latent period (12-24 hours, phase 3). During this period, asymptomatic hepatic and renal insufficiency may be developed (Garcia et al., 2015[42]). Weakness, overall deterioration, and liver necrosis are the first signs of the fourth and most dangerous phase. This stage is marked by fast central nervous system degeneration, intravascular coagulation, and severe hemorrhagic symptoms, including disseminated intravascular coagulation and renal failure. Encephalopathy and coma are symptoms of liver failure. Hepatic and renal failure are the most common causes of death in patients. Death can occur up to 6-8 days after the mushroom ingestion (Alves et al., 2001[2]; Garcia et al., 2015[42]). Furthermore, the presence of cardiovascular history and other comorbidities might increase the risk of sequelae and even death following amatoxin toxicity (Lecot et al., 2023[62])

#### Diagnosis and treatment

The time duration between ingestion and the start of symptoms, as well as the kind of systemic involvement (for example, neurologic vs gastrointestinal consequences), might assist the type of mushroom poisoning identification (Garcia et al., 2015[42]). As a result, the initial step is to assess symptomatology. The start of severe gastrointestinal symptoms after a delay of 8-24 hours after eating mushrooms suggests amatoxin poisoning. The latent phase is a defining feature of amatoxin poisoning. The presence of α-amanitin in urine confirms the diagnosis, which may be determined using a range of methods, including radioimmunoassay (RIA), enzyme-linked immunosorbent assay (ELISA), and high-performance liquid chromatography (HPLC) (Garcia et al., 2015[44]; Mas, 2005[70]).

For the analysis of amatoxins in urine on an emergency basis, RIA techniques have been developed. In some cases, such testing may be useful for confirming or excluding the diagnosis of amatoxin poisoning. However, 36 hours post-ingestion, this approach may produce erroneous negative results (Karlson-Stiber and Persson, 2003[57]).

The Meixner test can be used if a sample of the mushroom consumed is available for examination (Beutler and Vergeer, 1980[15]). The test is based on the formation of a blue product from an acid-catalyzed interaction of amatoxins with the complex biopolymer lignin. Lignin is a component of wood pulp that is removed during the conversion of pulp to high-grade cellulose paper but persists in lower-grade cellulose paper, such as newsprint. The test is performed by expressing the juice from a piece of fresh mushroom tissue onto a piece of newspaper, allowing the area to dry, and then adding one drop of strong hydrochloric acid. A positive test is when a blue hue appears (Beutler and Vergeer, 1980[15]). Although the Meixner test has a high detection limit for dangerous levels of alpha-amanitin, it fails to discriminate between alpha-amanitin and other mushroom indoles (Beuhler et al., 2004[14]).

Treatment of poisonings caused by amatoxin-containing mushrooms includes purgatory measures to eliminate any remaining toxic product from gastrointestinal tract and prevention of further toxin reabsorption via enterohepatic circulation (gastric lavage, activated charcoal, laxative, nasogastric/duodenal intubation and drainage, and cholestyramine); competitive inhibition of protein binding and cellular uptake of the toxin (antidotes); extracorporeal dialytic techniques to remove the toxin and replace coagulation deficits and orthotopic liver transplantation in cases of fulminant liver failure (Alves et al., 2001[2]).

The treatments employed for amatoxin poisoning are nonspecific, with none of them effectively reversing amatoxin-related hepatoxicity (Diaz, 2018[27]). Several antidotes are used, such as hormones (insulin, growth hormone, glucagon) steroids, vitamin C, vitamin E, cimetidine, thioctic acid, antibiotics (benzylpenicillin, ceftazidime), N-acetylcysteine, and silybin. From these, only thioctic acid, benzylpenicillin, ceftazidime, N-acetylcysteine, silybin, and cimetidine have been used in the pharmacologic management of amatoxin poisoning with varying success (Enjalbert et al., 2002[33]).

## Antidotes

### β-lactam antibiotics 

**Figure 8:**
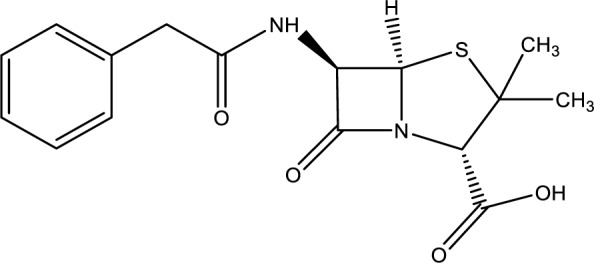
Chemical structure of Benzylpenicillin

As monotherapy or in combination with other drugs, benzylpenicillin (Figure 8(Fig. 8)) and other β-lactam antibiotics have been the most commonly used drugs in the treatment of amatoxin poisoning. However, the US Food and Drug Administration (FDA) has not authorized benzylpenicillin for the treatment of amanita poisoning (Enjalbert et al., 2002[33]).

The ability of benzylpenicillin to block amatoxin from entering hepatocytes and to directly compete with it for transmembrane transport has previously been proven (Letschert et al., 2006[64]).

Tong et al. (2007[105]) compared different treatments, including benzylpenicillin, to reduce liver damage in a mouse model of amatoxin-induced liver toxicity. They injected 0.6 mg/kg a-amanitin into six groups of mice over 48 hours. After 4 hours, the mice were randomly assigned to receive one of five intraperitoneal treatments (N-acetylcysteine, benzylpenicillin, cimetidine, thioctic acid, or silybin). These treatments were repeated every 4 to 6 hours. After 48 hours, the mice were sacrificed, and their liver enzyme levels (alanine transaminase and aspartate transaminase) were measured from aspirated cardiac blood. Liver samples were also collected to assess the extent of liver damage. The results suggested that benzylpenicillin was not effective in limiting hepatic injury after a-amanitin poisoning. Increases in aminotransferases and hepatonecrosis were not attenuated by this antidote (Tong et al., 2007[105]).

A therapeutic approach combining benzylpenicillin infusions with traditional support measures was administered to 33 severe A. phalloides poisoning cases in humans, resulting in a 100 % survival rate. Moroni et al. (1976[73]) consider benzylpenicillin a novel method capable of entirely altering the outcome of this mushroom poisoning when used appropriately alongside standard treatment (Moroni et al., 1976[73]).

Magdalan et al., (2010[67]) studied the effects of benzylpenicillin, N-acetylcysteine, and silybin as antidotes in human hepatocytes exposed to α-amanitin. They cultured primary hepatocytes for 48 hours with daily doses of α-amanitin (2 μM) and/or different concentrations of antidotes. Cell cytotoxicity assessment was conducted at 12, 24, and 36 hours of exposure to α-amanitin and/or antidotes. The findings demonstrated that N-acetylcysteine, benzylpenicillin, and silybin incubation in human hepatocyte cultures exhibited a similarly robust protective effect against α-amanitin-induced cell damage (Magdalan et al., 2010[67]).

In another study, benzylpenicillin's effectiveness was compared to ceftazidime and rifamycin SV in a human hepatocyte model. Primary hepatocyte cultures were maintained for 48 hours with daily doses of α-amanitin (2 μM) and/or the tested antidotes. Cytotoxicity assessment of the cultured cells was conducted after 48 hours of exposure to α-amanitin. The study's results provide proof that co-incubation of α-amanitin and benzylpenicillin effectively shielded human hepatocytes. Additionally, the study revealed that benzylpenicillin exhibited superior efficacy compared to ceftazidime and rifamycin SV as an antidote against α-amanitin (Magdalan et al., 2009[68]).

Patients on triple chemotherapy using MDAC, NAC, and penicillin G had the lowest mortality rate (8.3 %) among all assessed treatment plans (Trakulsrichai et al., 2017[111]).

**Figure 9:**
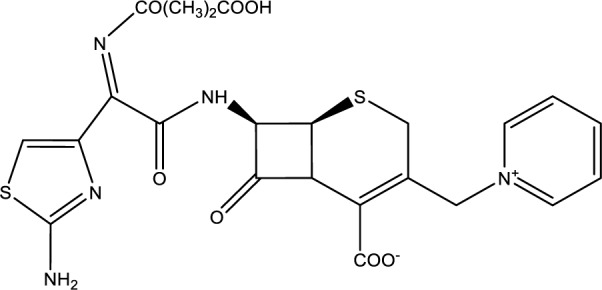
Chemical structure of Ceftazidine

Ceftazidine (Figure 9(Fig. 9)), a third-generation cephalosporin, has been significantly more effective than benzylpenicillin with fewer side effects. This antidote was the second most commonly used β-lactam, always in conjunction with silybin (Enjalbert et al., 2002[33]).

### Silymarin

**Figure 10:**
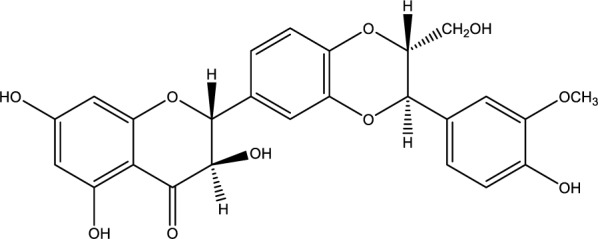
Chemical structure of Silybin

*Silybum marianum* (milk thistle) is the most extensively studied plant for treating liver disease. Its active compounds, such as silybin (Figure 10(Fig. 10)), silydianin, and silychristine, collectively called silymarin, are flavonolignans. Among them, silybin exhibits the highest biological activity, and milk thistle extracts are typically standardized to contain 70-80 % silybin (Luper, 1998[65]).

Silymarin's protective effects were examined in various experiments. Dogs received lethal doses of *A. phalloides* toxin. Administration of silybin (50 mg/kg) at 5 and 24 hours after poisoning mitigated serum changes (including Glutamyl oxaloacetic transaminase, Glutamyl pyruvic transaminase, alkaline phosphatase, and bilirubin) and prevented a decline in prothrombin time. The extent of hemorrhagic liver necrosis was significantly diminished (Vogel et al., 1984[113]).

The hepatoprotective action of silymarin was demonstrated by Desplaces et al., (1975[26]). Their research confirmed that an administration of 15 mg/kg of silymarin to animals (such as dogs, rabbits, rats, and mice) protected all animals when given 60 minutes before the toxin. Likewise, injecting silymarin at a dose of 100 mg/kg 10 minutes after *A. phalloides* exposure also resulted in complete protection (Desplaces et al., 1975[26]). However, in a murine model silybin was not effective in limiting hepatic injury after α-amanitin poisoning (Tong et al., 2007[105]). As previously described, Magdalan et al., (2010[67]) demonstrated that administering benzylpenicillin, N-acetylcysteine, and silybin to human hepatocyte cultures exhibited a similarly potent protective effect against amanitin toxicity. The cytoprotective effect of these antidotes wasn't dependent on the dose, indicating their high effectiveness (Magdalan et al., 2010[67]).

A total of 18 cases of *Amanita palloides* intoxication were treated by combined chemotherapy (Hruby et al., 1983[54]). All patients received silybin as standard treatment. It was noted that administering silybin even up to 48 hours after consuming the mushroom was an effective measure in preventing severe liver damage from amanita poisoning (Hruby et al., 1983[54]). Similarly, oral administration of silymarin was effective in 87.5 % of poisoning patients with hepatitis diagnosis (Trakulsrichai et al., 2017[111]).

The mechanism of action of silybin is still poorly understood. Silymarin and silybin hinder the absorption of toxins like phalloidin or α-amanitin, stopping them from attaching to cell surfaces and blocking membrane transport systems (Fraschini et al., 2002[37]).

Moreover, silymarin and silybin can alter the chemical and physical properties of cell membranes by interacting with their lipid components. Additionally, the proven ability of silymarin and silybin to neutralize free radicals can clarify their protective effects against liver-damaging substances. These antidotes likely work by countering free radicals and disrupting the lipid peroxidation processes implicated in liver injury caused by toxins. They may counteract the decline of two key antioxidants, glutathione (GSH) and superoxide dismutase (SOD), by reducing the burden of free radicals, boosting GSH levels, and enhancing SOD activity (Fraschini et al., 2002[37]).

Silymarin can penetrate the nucleus and influence RNA polymerase enzymes, leading to higher ribosomal production. This action holds significant therapeutic potential for mending harmed hepatocytes and reinstating normal liver functions (Pradhan and Girish, 2006[81]).

### Thioctic acid

**Figure 11:**
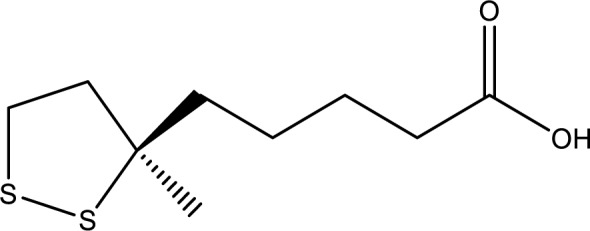
Chemical structure of Thioctic acid

Thioctic acid (Figure 11(Fig. 11)), also known as α-lipoic acid, is an antioxidant compound. It collaborates with antioxidants such as vitamins C and E. Unlike other antioxidants, it possesses unique qualities that suggest it might be capable of substituting specific nutritional supplements. It promotes growth, aids energy production, and assists the liver in detoxifying the body. Thioctic acid also shields cells, regulates blood sugar and eliminates toxic metals from the bloodstream (Roy et al., 2021[93]).

Studies on thioctic acid's mechanism propose its potential for treating amatoxin hepatotoxicity. It acts as a free radical scavenger and could inhibit cell membrane lipid peroxidation by releasing hydrogen ions from dihydrothioctic acid, its primary metabolite (Enjalbert et al., 2002[33]).

The Instituto Seroterapico Italiano in Milan suggested that up to 300 mg of thioctic acid could be safely administered in a day through continuous infusion (Steyn, 1966[102]). Thirty-nine out of 40 patients were saved by this treatment in Ceske Budejovice in Bohemia (Steyn, 1966[102]). Thioctic acid infusions were used in the treatment of amanita mushroom poisoning in 75 patients between 1974 and 1978. While 10-50 % of patients recover without intervention, 89 percent (67 of 75) recover after lipoic acid infusion (Berkson, 1979[12]) A case involving a 32-year-old man who consumed *A. phalloides* highlighted thioctic acid's potential efficacy. Intravenous infusion of 300 mg within 24 hours, mixed with 10 percent glucose in water, led to the patient's improvement and symptom resolution on the following day (Becker et al., 1976[10]).

However, Poucheret et al., (2010[80]) reported that thioctic acid didn't significantly impact survival rates and showed limited therapeutic effects in the context of amatoxin poisoning. The lack of significant impact on patient survival by this compound may stem from its free radical scavenging effect not primarily targeting the main site of acute oxidative stress in amatoxin poisoning, which is the liver. Thioctic acid is known for potential side effects like hypoglycemia, which could worsen glucose imbalance induced by amatoxins. Additionally, it stimulates COX (cyclooxygenase) activity and related prostaglandin production, potentially conflicting with potent drugs like silybin if used together in treatments. These factors collectively contribute to thioctic acid's controversial role in amatoxin poisoning treatment (Poucheret et al., 2010[80]).

### N-acetylcysteine

**Figure 12:**
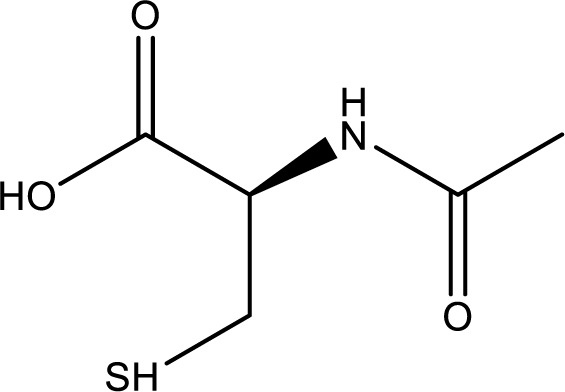
Chemical structure of N-acetylcysteine

N-acetylcysteine (Figure 12(Fig. 12)) has been utilized in medicine for over 50 years as a mucolytic agent. It is recognized for addressing acetaminophen overdose and associated liver failure. Yet, N-acetylcysteine (NAC) serves as a versatile supplement, serving as a precursor to glutathione and elevating overall body glutathione levels (Tenório et al., 2021[103])

Acetaminophen transforms into harmful free radicals in the liver, binding to glutathione. NAC serves as a glutathione precursor and works as an effective therapy for acetaminophen overdose. Testing in mice indicated that NAC might not have a clinical role in treating *A. phalloides* ingestion, as its administration did not impact survival or liver enzyme elevation when compared to control animals (Schneider et al., 1992[97]). On the other hand, Magdalan et al., (2010[67]) demonstrated that exposing human hepatocyte cultures to NAC exhibited potent protection against α-amanitin-induced cytotoxic damage (Magdalan et al., 2010[67]).

According to Poucheret et al. (2010[80]), NAC operates on two levels. Firstly, it scavenges free radicals in a general manner. Secondly, its more targeted effect involves replenishing liver glutathione. Furthermore, NAC could potentially disrupt the intramolecular tryptathione bridge of amatoxin (critical for its toxicity). NAC administration is primarily associated with hepatoprotective function rather than an antidote role (Enjalbert et al., 2002[33]). A retrospective study found that the most frequently reported treatment (69.9 %) for amatoxin-intoxicated patients involved a combination of NAC and silybin (Lecot et al., 2023[62]). Consequently, further research is needed to thoroughly validate these mechanisms of action for NAC.

### Cimetidine

**Figure 13:**
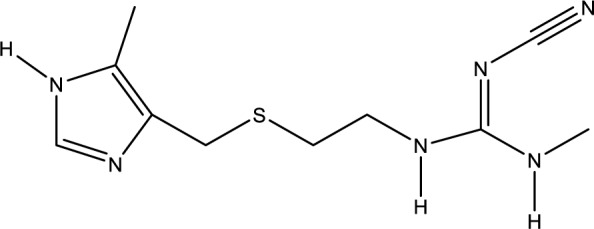
Chemical structure of Cimetidine

Cimetidine (Figure 13(Fig. 13)), a treatment typically used to reduce stomach acid production, has been studied for its ability to inhibit the uptake of amatoxins in the liver. By doing so, it may help mitigate the toxic effects of these compounds on the liver cells (Bang et al., 2022[7]) 

As a cytochrome P450 inhibitor, it was initially applied in amatoxin exposures due to the resemblance between amatoxin poisoning and other liver toxins that affect cytochrome P450 (Tong et al., 2007[105]).

Schneider et al. (1987[96]) studied cimetidine as an antidote against the key mushroom toxin α-amanitin. Examination of liver tissues from α-amanitin poisoned mice displayed significant mitochondrial alterations, but the hepatic mitochondria remained intact in mice treated with cimetidine either before exposure or within 6 hours (Schneider et al., 1987[96]). 

However, it is important to note that while there is some experimental evidence suggesting cimetidine's potential benefits in amatoxin poisoning, further clinical research is needed to establish its effectiveness and safety in human cases.

### Promising putative antidotes

**Figure 14:**
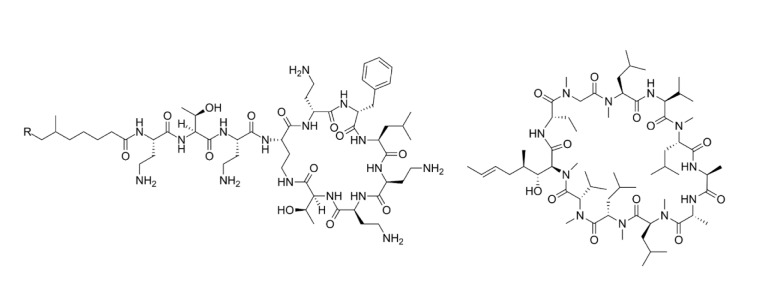
Chemical structure of Polymyxin and Cyclosporine, respectively

In addition to established treatments for amatoxin poisoning, two potential promising antidotes have emerged: Polymyxin B (Pol) and cyclosporine A (CsA) (Figure 14(Fig. 14)). Experimental studies have shown that α-amanitin-intoxicated mice treated with Pol fully recover from toxicity, displaying reduced cellular degeneration and necrosis in the liver and kidney (Garcia et al., 2015[44]). When combined with methylprednisolone, Pol also lowered α-amanitin-induced liver damage, resulting in 100 % survival of treated animals (Garcia et al., 2019[41]). The suggested mechanism for Pol's effectiveness is its binding to RNAP II, thereby preventing α-amanitin from binding to the enzyme (Garcia et al., 2015[44]). However, *in vitro* studies did not observe any effects of Pol on α-amanitin cytotoxicity in cultured HepG2 cells (Rodrigues et al., 2020[91]) and there is currently no evidence for the efficacy of this potential antidote in humans intoxicated with α-amanitin.

On the other hand, CsA has also been explored for its potential to inhibit the OATP1B3 transporter, which prevents α-amanitin uptake by hepatocytes, thus averting hepatoxicity (Mackenzie et al., 2022[66]). Experimental animals treated with CsA showed hepatoxicity recovery, with 40 % survival when receiving CsA injections 4, 8, and 12 hours after α-amanitin exposure, and 100 % survival with simultaneous treatment. Additionally, CsA treatment reduced liver edema, necrosis, and cytoplasmic vacuolization (Garcia et al., 2022[39]). In human cases, a series of reports indicated that CsA administration (5 mg/kg IV over 4-6 hours every 24 hours) was effective in treating amatoxin ingestion in three patients. Moreover, the two patients with elevated aminotransferase levels fully regained normal liver enzyme values after CsA treatment (Mackenzie et al., 2022[66]).

### Transplantation

Acute liver failure (ALF) is a severe syndrome where previously healthy individuals experience sudden and serious liver cell dysfunction. This can quickly lead to coma and death because of cerebral edema and failure of multiple organ systems (Larson 2008[61]). Nonetheless, not all *A. phalloides* poisoning patients experience acute liver failure, and not all cases result in fatality (Escudié et al., 2007[34]).

There are two surgical choices for liver transplantation: orthotopic liver transplantation and auxiliary liver transplantation. Orthotopic liver transplantation necessitates ongoing immunosuppression to sustain the graft. Auxiliary partial liver transplantation is an option when certain native livers can recover with partial hepatectomy and temporary support. The transplanted liver then offers temporary aid until the native liver regenerates (Barceloux, 2008[8]).

Timely and precise assessment of an individual patient is crucial in determining whether liver transplantation is necessary for treating fulminant hepatic failure. The Liver Unit at King's College Hospital has devised a prognostic model to identify patients with poor prognosis. The criteria for non-acetaminophen-induced fulminant hepatic failure include: prothrombin time > 100 seconds; any three of the following: age <10 or >40 years, jaundice >7 days before encephalopathy onset, prothrombin time >50 seconds, and bilirubin >300 μmol/L (O'Grady et al., 1989[77]). Furthermore, Escudié et al. (2007[34]) recommended that patients with an interval of <8 hours between ingestion and diarrhea should strongly consider liver transplantation. Encephalopathy need not be an absolute prerequisite for transplantation decision. Starting from day 4 of ingestion, a prothrombin index lower than 10 % alone is a reliable indicator for urgent transplantation (Escudié et al., 2007[34]). 

Although definitive predictors are lacking, factors like encephalopathy stage, coagulation tests, metabolic irregularities, and age can aid in the strategic planning of liver transplantation (Enjalbert et al., 2002[33]). 

### Special populations

#### Pregnant patients 

A few case reports document the consumption of amatoxin-containing mushrooms during pregnancy. In one instance, a 25-year-old woman consumed *A. phalloides* at 9 weeks gestation, leading to poisoning. She survived and underwent a therapeutic abortion at 12 weeks. The fetus exhibited hepatocellular damage, indicating that amatoxin could cross the placental barrier. 

Conversely, certain studies indicate that amatoxins don't traverse the placental barrier (Belliardo et al., 1983[11]), even in the acute phase of poisoning. As a result, their harmful impact on fetal nucleic acids and proteins wouldn't occur, explaining the absence of fetal harm. A documented case describes a woman surviving *A. phalloides* ingestion without fetal damage or impairment to the child's growth and development. Consequently, even in the first trimester of pregnancy, maternal poisoning by *A. phalloides* might not necessarily warrant abortion (Boyer et al., 2001[19]).

## Conclusion

Numerous toxic reactions caused by wild mushrooms have been documented in the literature, some leading to severe or fatal consequences. These include brain edema, liver necrosis, and kidney damage from *Gyromitra esculenta* ingestion; acute renal failure from *C. orellanus* ingestion; convulsions and coma from *Amanita pantherina* ingestion; and combined liver and renal failure from *A. phalloides* ingestion.

Some mushrooms, such as *Coprinus atramentarius*, *Psilocybe cubensis*, *Amanita muscaria*, and *Chlorophyllum molybdites*, are recognized as toxic but are associated with limited or no human fatalities. However, their consumption can lead to prolonged morbidity and disease progression.

Toxins like gyromitrin, orellanine, and amatoxins are characterized by a latent period before the onset of illness, while gastrointestinal symptoms predominate in the groups exposed to amatoxins, gyromitrin, and GI irritants. Mushrooms containing psilocybin, ibotenic acid, and muscimol induce hallucinogenic and neurological effects when ingested. Muscarine-containing mushrooms trigger an acute peripheral cholinergic syndrome, and coprine toxicity arises only when consumed alongside alcohol.

Mushrooms containing cyclopeptides are highly toxic and account for the majority of fatalities resulting from mushroom consumption. The exact mechanism of liver damage remains incompletely understood. Some researchers suggest that amatoxins hinder RNA polymerase II activity, leading to reduced mRNA levels, which in turn disrupts protein synthesis and triggers cell death, particularly in the liver. Oxidative stress has also been proposed to enhance the toxicity of these toxins, although the specific pro-oxidant mechanisms are not yet identified.

Various antidotes have been provisionally utilized in cases of amatoxin poisoning. These include hormones (insulin, growth hormone, glucagon), steroids, vitamin C, vitamin E, cimetidine, α-lipoic acid, antibiotics (benzylpenicillin, ceftazidime), N-acetylcysteine, and silibinin. Among these, only thioctic acid, benzylpenicillin, ceftazidime, N-acetylcysteine, silibinin, and cimetidine have been employed in the pharmacological treatment of amatoxin poisoning in humans, yielding different degrees of success.

Benzylpenicillin and other β-lactam antibiotics are commonly employed for treating amatoxin poisoning alone or alongside other medications. Yet, the specific mechanisms by which these drugs work are still unclear. In cases of liver failure, liver transplantation might be the sole viable option.

Mistaking toxic mushrooms for edible ones during wild mushroom consumption is the primary cause of mushroom poisoning. The best prevention against mushroom poisoning is abstaining from eating wild mushrooms. In general, distinguishing between non-poisonous and poisonous mushrooms is challenging for individuals without expertise in mushroom identification.

In conclusion, it is essential to advance our understanding of the toxic mechanisms caused by these toxins, particularly amatoxins. Such knowledge is pivotal for developing improved and more efficient antidotes.

## Notes

Irene Gouvinhas and Juliana Garcia (AquaValor – Centro de Valorização e Transferência de Tecnologia da Água – Associação, Rua Dr. Júlio Martins n.º 1, 5400-342 Chaves, Portugal; E-mail: juliana.garcia@aquavalor.pt) contributed equally as corresponding author.

## Declaration

### Funding

This work is supported by National Funds by FCT-Portuguese Foundation for Science and Technology, under the project UIDB/04033/2020 (https://doi.org/10.54499/UIDB/04033/2020) (accessed on 2 April 2024).

### Conflict of interest

The authors declare that they have no conflict of interest.

### Acknowledgments

Irene Gouvinhas thanks FCT (Fundação para a Ciência e Tecnologia) for funding through the Scientific Employment Stimulus-Individual Call (2022.00498.CEECIND), https://doi.org/10.54499/2022.00498.CEECIND/CP1749/CT0001 (accessed on 2 April 2024). Juliana Garcia and Jani Silva are grateful for funding support from Portuguese public funding through Investimento RE-C05-i02-Missão Interface N.º 01/C05-i02/2022 and from FCT for the projects titled “AquaValor-Centro de Valorização e Transferência de Tecnologia da Água” (NORTE-01-0246-FEDER-000053), supported by Norte Portugal Regional Operational Programme (NORTE 2020), under the PORTUGAL 2020 Partnership Agreement, through the European Regional Development Fund (ERDF).

## Figures and Tables

**Figure 1:**
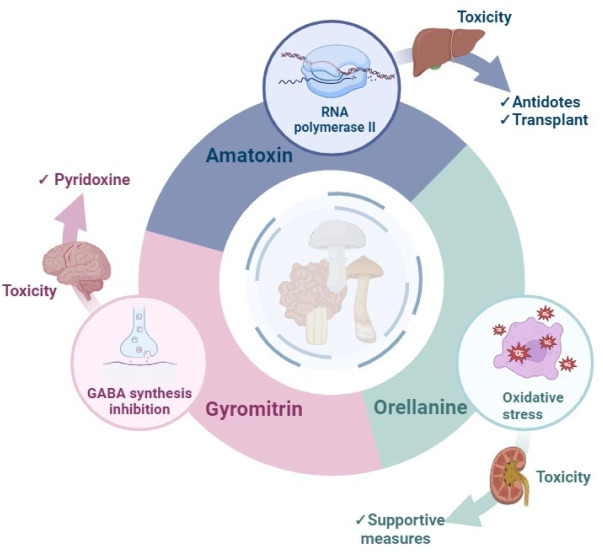
Graphical abstract
